# Drafts

**Published:** 1871-10

**Authors:** Ben. H. Pratt


### DRAFTS.

BEN. H. PRATT, A. M.

That incongruities in Nature should so much abound, is strange. That the popular mind should allow itself to be confounded by them—utterly non-plussed by them, is not at all singular.

To a quiet observer, idiosyncrasies are a study.

Take, for instance, the theory of Drafts. By Drafts, in the present case, I mean currents of air. If there is any one subject upon which folks are eminently sensible, it is upon this.

Public sentiment is almost unanimous in ascribing the majority of bodily ailments to Drafts.

If James coughs, or John sneezes, or William snores, or Peter has a cold in his head, or Jacob has rheumatism, of course my dear boy has been or is being exposed to a current of air, somehow, somewhere; and a denial of the parental fiat is as monstrous as it is imprudent.

What a perfect haven of bliss this earth would be if it were not for those direful Drafts that are eternally shoveling us into sick beds, and thence into early graves. How differently most of us would have ordained it had we been consulted in the economy [illegible] Nature.

In much, nay, in almos[illegible] every particular, Nature is perfection in all her workings; but in the matter of which we now speak, how signal the failure.

The gentle zephyrs that sway the leafy tree tops so gracefully—that cause the undulations of the ripening grain in countless fields, which all admire—that stud the deep with billowy beauty and cause its wavelets to beat the beach and sprinkle beads of glory on the silvery shore—that fill the sails of countless vessels, bearing creature comforts to every clime—that twirl the spreading fans of thousand windmills, blessing many a hamlet with its rich results of snowy, healthful, life-supporting meal—that bear from far the balmy sweets of flowery meads to thrill our senses with ecstatic joy—that lift from moor and fen and fever-breeding marsh, and bear far from our sphere the pestilential airs which there instill—that creep on silent errands into every nook and corner of our homes, and cleanse them all of every noxious deleterious breath, that we may breathe the purest air and live—these are but trifling benefits, compared with all the ills to which the Draft gives rise. But so it is; and being so, what's to be done? Ignore the Draft and die? That's not agreeable to contemplate. Avoid all Drafts as soon as recognized? Well, yes; but then it's very inconvenient so to do, and oft times impolite, and then indeed it's sometimes such a weakness to so fear a Draft, lest some benighted unbeliever in its power may ridicule our prudence. That too is very humiliating.

Alas! we're at a loss what to advise. To what discomforts—what unrest—what misery—what perplexity the Draft subjects us.

Invited out to dine, a seat is given us perhaps between two windows; and the summer breeze enters, and we feel its hateful breath upon our neck and back. If we are bold, we rise and close the sash instanter, and the master or mistress of the feast smiles sweet compliance with our act—but when we're gone, rains down upon our absent head a bitter shower of abuse at our attempt to regulate the affairs of those whom we may visit—calls it impudence and downright meddling—and vows that we shall never poke our legs beneath their groaning mahogany again. That's very sad. If, however, we are of the timid sort, and much inclined to submit and take the consequences, then our misery begins. We endure awhile and finally look behind us, hoping our glance may call attention to the cause of our discomfort. But in vain. Soon we begin to wiggle as do restless children at their meals, and then display our misery in countless ways, until our hosts are well aware that we are ill at ease; and all unconscious of the cause, begin surmises.

Now, of all the ills my neighbor can do me, I do think surmises breed the worst. What won't surmising beget! Your peculiar behavior at your friend's table during the course of one brief meal, may result in blasting your reputation forever. Your host nudges his next at table and attention is called to you. After dinner, two heads, mayhap two pairs of heads, get close together to consult upon your case, and be assured that nothing good can be the result. There's such a latitude for the surmiser. Of all the things I pray to be relieved from, it is from the surmises of my friends.

In a company you cross the room to speak to, or have a cozy chat with some particular friend of the other sex, and having just entered into the real enjoyment of the same, you suddenly perceive that an open window is near you. Suddenly falls the thermometer of all your pleasant anticipations of a joyous time. To be or not to be—to suffer or retreat—these are the questions to be promptly answered; and upon the issue hang a multitude of sequences. No doubt that many a prudent belle has gone husbandless to old maidenhood—that many a dear fellow has doled out years of wifeless misery from a single Draft. How cruel, this, of dame nature, whom poets praise and philosophers worship.

What makes the matter ten-fold worse, is the fact that in every community there exist individuals upon whom the Draft seems to have no ill effects whatever.

I say *seems;* for we sensitively organized, delicately reared, nervously tempered people, know that they are only great senseless blocks of humanity, or else possess some secret charm, some potent agent that antidotes the Draft. They sneeze occasionally and perhaps get colds now and then, but live provokingly long, and have provokingly few serious ills, and are provokingly comfortable, nay even happy, most of the time. These are they who sneer. They also are much given to ridicule. They laugh at the death dealing Draft until it looks like unto a defying of Providence. They shudder perhaps at the earthquake, and flinch a little at the lightning's stroke when nigh, but utterly fail to see the far more terrible havoc which steals on the silent wings of the Draft. They sleep beneath an open window with no consciousness of harm. They defy with impunity the stealthiest Draft that ever wooed an unconscious wretch to his tomb.

Another feature of the Draft is its baneful effects at one time, and its absolute innocence at another. I know of certain ones upon whom a most pernicious Draft (to all appearances) had actually played for hours. The escape was impossible and naught could be done but submit, and the unhappy victims patiently, nay almost calmly awaited its fatal effects. Wonderful as it may seem, they escaped all signs of ill, and chagrined beyond measure, detailed the astonishing result to astounded friends, and lived to tell the tale to their great grand children. To account for the fickle character of this scourge, has baffled the wisdom of philosophers and sages the world over. Perhaps the world will never live to see the solution of this seeming inconsistency in Nature.

It is still more improbable that we shall ever so far advance in the investigation of the subject as to be able correctly to distingush between the Draft fatal and the Draft innocuous. Could this be attained, however, how great a boon would a now afflicted world possess.

In imagination we can behold a future generation to which such wisdom shall have come, and musing thereon, contemplate a race of perfectly happy beings, whom no fears of Drafts shall menace, nor any uncertainty provoke. Should they by some dire chance confront the Draft fatal, then they can lie down in blissful certainty of its effects; while the Draft innocuous will scarce deserve a thought, save to recall the ignorance of their ancestors which led them to fear it; just as we now lament the witch-killing propensity which ignorance imposed upon our otherwise model forefathers.

Then, again, we poor mortals can see no earthly difference between a Draft and a wind. Draft is wind, and wind is Draft. But we face the wind—back the wind—do all sorts of imprudent things with the wind. It's only when it tumbles us up and tips us over, and musses our hair, and sends signs and shutters and branches of trees and bricks, and other objectionable missiles, about our ears, that we entertain fear--actual alarm.—But a little breeze, coming in at the window, or beneath the door, scarcely perceptible, sometimes unnoticed, sets us to ailing and dosing and grumbling, and otherwise making ourselves and others uncomfortable and sick.

As I remarked in my first sentence, so I will remark in this the last: Incongruities in nature do much abound, and it is strange.

Yes, it's very, very strange—TO SOME.

